# Buffalo Ophthalmological Club

**Published:** 1902-05

**Authors:** 


### Buffalo Ophthalmological Club.

A new ophthalmological club in Buffalo was organised March 14, 1902, at the office of Dr. Benjamin H. Grove, 334 Pearl Street, by the following gentlemen: Drs. Alvin A. Hubbell, Benj. H. Grove, Lucien Howe, Arthur G. Bennett, F. Park Lewis, Lorenzo Burrows, Jr., Richard H. Satterlee, John J. Finerty, John C. Clemesha, Albert E. Hubbard, John D. Flagg, Elmer G. Starr, Henry Y. Grant and Benjamin F. Rogers.

The name of the organisation is the Buffalo Ophthalmological Club.

At this initial meeting by-laws were formulated and officers elected. The officers are an executive committee, consisting of Drs. Benj. H. Grove and Lucien Howe, and a secretary-treasurer, Dr. John D. Flagg.

The meetings, which are held the second Thursday evening of each month from September to May inclusive, are both professional and social.

The first regular monthly meeting of the club was held Monday evening, April 14, 1902, at the home of Dr. F. Park Lewis, 454 Franklin Street.

At this meeting, Dr. Grove presented a patient with a small piece of iron in the vitreous of the left eye. It had been in the eye five months and was encysted in what appeared to be lens substance. As the eye gave the man no particular trouble he refused to have it removed.

Dr. John J. Finerty presented a patient with a choroidal exudate near the macula in the right eye. The patient could give no history of injury except that about two months ago a drop of oil flew in the eye.

Dr. Arthur G. Bennett exhibited two specimens, an ossified choroid and a calcified lens, both removed from the same eye.

Dr. A. A. Hubbell made some appropriate remarks in introducing the subject, Astigmatism and its correction.

The other members present took part in the discussion, after which refreshments were served and a social hour thoroughly enjoyed.

The club then adjourned to meet the second Thursday in May at the home of Dr. Lorenzo Burrows, Jr.

